# Oils extracted from *Eupatorium adenophorum* leaves show potential to control *Phythium myriotylum* in commercially-grown ginger

**DOI:** 10.1371/journal.pone.0176126

**Published:** 2017-05-03

**Authors:** Xiaoman Liu, Dongdong Yan, Canbin Ouyang, Dongsheng Yang, Qiuxia Wang, Yuan Li, Meixia Guo, Aocheng Cao

**Affiliations:** 1 Institute of Plant Protection, Chinese Academy of Agricultural Sciences, Beijing, China; 2 Institute of Environment and Sustainable Development in Agriculture, Chinese Academy of Agricultural Sciences, Beijing, China; Gyeongnam National University of Science and Technology, REPUBLIC OF KOREA

## Abstract

Oils extracted from the leaves of *Eupatorium adenophorum* were tested *in vitro* and *in vivo* against the soilborne pathogen *Pythium myriotylum* which causes soft rot, a devastating disease of commercial ginger production in China. Twelve compounds accounting for 99.15% of the total oil composition were identified by GC-MS. The major components were 10Hβ-9-oxo-agerophorone (37.03%), 10Hα-9-oxo-agerophorone (37.73%) and 9-oxo-10, 11-dehydro-agerophorone (23.41%). Antifungal activity was tested by the poisoned food technique against *P*. *myriotylum*, indicating minimum inhibitory concentrations of 100μg/ml after 7 days incubation. In addition, the oil extracts greatly inhibited the formation of both wet and dry mycelial biomass. The combination of *E*. *adenophorum* oil extracts and synthetic fungicides showed a strong synergistic effect, inhibiting the mycelial growth in *in vitro* assays. The synergistic effect of oil extracts with fungicides could allow fungicides to be used at reduced rates in the future which has environmental advantages. Oil extracts applied at 160 and 200μg/ml concentrations to ginger rhizomes before inoculation with *P*. *myriotylum* significantly reduced the infection rate in ginger. Examination by light and transmission electron microscopy revealed that oil extracts caused swelling of the hyphae, disruption of the cell wall, degradation of the cytoplasmic organelles and shortening of the cytoplasmic inclusion. These results suggested that the plasma membrane and endomembrane systems of *P*. *myriotylum* were severely damaged by the oil extracts of *E*. *adenophorum* which offer significant potential for use as a fungicide to control *P*. *myriotylum*.

## Introduction

Ginger (Zingiber officinale Rosc.) is an important cash crop which is widely planted in China, especially in Shangdong district. Ginger soft rot, caused by the soilborne pathogen *Pythium myriotylum*, is one of the most devastating diseases of ginger [1]. The first symptoms of infected ginger are yellow leaves and collapsed shoots; below ground, water-soaked lesions appear on the developing rhizome [2]. Under suitable conditions the rhizome rots rapidly and an infested field can be destroyed by the pathogen within a week [3].

*P*. *myriotylum* is a hemi-biotrophic organism belonging to the Oomycetes class. It is widespread in India, China, Japan, Nigeria, Fiji, Australia, Sri Lanka, Hawaii and Korea [4]. *P*. *myriotylum* is not only pathogenic to ginger but also to many other crops, such as cocoyam, bean, groundnut, tomato, tobacco and watermelon, resulting in significant decreases in yield and quality [5].

Historically, only a few compounds have been registered for the control of *P*. *myriotylum* in China. Mefenoxam is the most frequently used fungicide in ginger seed treatment, which is effective in controlling ginger soft rot. However, excessive use of chemical pesticides has polluted the environment and harmed human beings and animals. Moreover, excessive use has resulted in pathogen resistance to pesticides. Because of these concerns, researchers are seeking new sources of materials to control Oomycete pathogens in crop production. Plant extracts are among the sources being investigated. Plant products have received global attention because they have constituents with novel structures; they are produced naturally, are biodegradable and generally do not leave toxic residues or byproducts which contaminate the environment; and they have less potential for developing resistance to pathogenic microorganisms [6–8]. Moreover, many oil extracts from plants are classified as GRAS compounds (“generally regarded as safe”) by the widely-regarded United States Food and Drug Administration.

*Eupatorium adenophorum* Spreng is a strongly invasive plant originally from Mexico but now a globally-widespread malignant weed. *E*. *adenophorum* may have spread globally when free of its natural enemy constraints (such as competitors, pathogens and predators). It releases inhibitors which interfere with the development of other species [9, 10]. In recent years, an increasing number of studies have shown that the terpenes in *E*. *adenophorum* comprise a class of important inhibitors which have allelopathic activity, antioxidant activity, antimicrobial activity, acaricidal activity, nematode activity as well as activity against other pests [11, 12–17]. However, there are no known reports of the ability of *E*. *adenophorum* oil extracts to control *P*. *myriotylum*.

The present study identified the chemical composition of oil extracts from the leaves of *E*. *adenophorum* (termed ‘oil extracts’) and evaluated their effect against *P*. *myriotylum in vitro* mycelial growth and mycelial weight as well as *in vivo* activity. Moreover, its synergistic effects where assessed when mixed with synthetic fungicides on *P*. *myriotylum in vitro*. As a result of this work, a preliminary explanation was suggested for the possible mode of action of *E*. *adenophorum* oil extracts against the mycelium growth of *P*. *myriotylum*.

## Materials and methods

### Plant material

Leaves of *Eupatorium adenophorum* were collected from Sichuan Province of China in May 2014. The plant was identified by Prof. Aocheng Cao at the Institute of Plant Protection, Chinese Academy of Agricultural Sciences, Beijing. Voucher specimen no. 20060712 was deposited at the College of Chemistry, Beijing Normal University. No specific permission was required as the plant material was sourced from uncultivated land, and the field studies did not involve endangered or protected species.

### Extraction and isolation

The air-dried leaves were ground in a mill, and then passed through a mesh screen to obtain a uniform 40 mesh size. The powder was extracted with methanol for 12 h, and then treated by ultrasonic waves for 30 min at ambient temperature. The supernatant was evaporated to dryness under reduced pressure using a rotary evaporator. The crude methanol extract was dissolved in a small amount of methanol and extracted with ethyl acetate. The ethyl acetate extract was purified using XAD-2 macroreticular resin and eluted with MeOH: H_2_O: CHC1_2_ (85: 10: 5). The elution liquor was concentrated and subjected to column chromatography over silica gel (200–300 mesh) which was first eluted with dichloromethane to remove non-polar compounds, followed by a mixture of dichloromethane: ethyl acetate 98: 2. The eluent was collected and found to be effective against *P*. *myriotylum*. After freeze-drying, a pale-yellow oily product was stored in an airtight sealed glass vial at 4°C for further testing. The yield of the oil extracts was 0.92%.

In addition, two oil extracts namely 9-oxo-agerophorone and 9-oxo-10, 11-dehydro-agerophorone were isolated from *E*. *adenophorum* using methods described previously [18, 19]. The purity of two compounds for the antifungal tests was over 90%.

### Gas chromatography-mass spectrometry (GC-MS) analysis conditions

The oil extracts were subjected to GC-MS analysis for identification of their chemical composition. GC-MS analysis was performed using a Thermo Scientific ISQ single quadrupole GC-MS, equipped with HP-5 MS capillary column (30m x 0.25mm x 0.25μm). For GC-MS detection, an electron ionization system was used (70eV ionization energy). The carrier gas was helium at a flow rate of 1 ml/min. Injector, ion source and MS transfer line temperatures were set at 250°C. The column temperature was initially kept at 50°C for 1 min, and then gradually increased to 280°C at a rate of 10°C/min, with a final 5 min of heating at 280°C. The injector volume was 2 μl in splitless mode. The chemical components were identified by comparing their relative retention time and mass spectra with those of standards and NIST library data of the GC—MS system.

### Fungal strains used

*Pythium myriotylum* was collected from field-infected ginger plants in Anqiu city, Shandong province, China. *P*. *myriotylum* was isolated, purified and maintained on a potato dextrose agar (PDA) medium at 28 ± 1°C.

### Antifungal effect of *E*. *adenophorum* oil extracts on mycelial growth

A poisoned food technique was used to evaluate antifungal activity against *P*. *myriotylum* [20]. The oil extracts and two compounds (9-oxo-agerophorone and 9-oxo-10, 11-dehydro- agerophorone) were prepared using dimethyl-sulfoxide (DMSO, 0.5% v/v) as the initial solvent carrier followed by dilution with PDA (at about 50°C) to produce the desired concentrations of 40, 60, 80, 100 and 120 μg/ml. A 4 mm mycelial disk was cut from the periphery of 2-day-old cultures, placed in the center of each PDA plate, and then incubated in the light-dark cycle at 28 ± 1°C for 7 days. PDA plates treated with an equal quantity of DMSO were used as a negative control. Each treatment was repeated in triplicate. The mycelial growth (mm) in both treated and control Petri dishes were measured diametrically in two different directions using a vernier caliper. The percentage inhibition of fungal growth was calculated using the following equation when the mycelial mass had almost filled the Petri dish in the control:

Inhibition(%)=[(C−T)/(C−4)]×100

Where C is the diameter of fungal colony in the control; T is the diameter of fungal colony in the treatment; and 4 is the diameter of the inoculum disc.

### Combined action of *E*. *adenophorum* oil extracts and commercially available fungicides

The effect of the mixtures on *P*. *myriotylum* was determined in radial growth experiments as described above. The treatment groups were: a) control group (PDA containing DMSO); b) *E*. *adenophorum* oil extracts in PDA; c) *E*. *adenophorum* oil extracts combined with a commercial fungicide (mefenoxam, mancozeb, iprodione or azoxystrobin) in PDA; and d) individual commercial fungicide in PDA. The interactive effect of the mixed components was evaluated using the formula provided by Colby [21, 22]:

$${\text{E}}_{\text{xp}}={\text{X}}_{\text{A}}{\text{Y}}_{\text{B}}/100$$

In this equation, E_xp_ = the expected mycelial growth inhibition rate of the mixture, X_A_ = mycelial growth inhibition rate of *E*. *adenophorum* oil extracts alone, Y_B_ = mycelial growth inhibition rate of commercial fungicides (mefenoxam, mancozeb, iprodione or azoxystrobin) alone, O_bs_ = the observed mycelial growth inhibition rate of the mixture. The synergism ratio (SR) is calculated as the ratio between O_bs_ and E_xp_. If SR = 1, there is additive; If SR > 1, there is synergism; If SR < 1, there is antagonism.

### Antifungal effect of *E*. *adenophorum* oil extracts on wet and dry mycelial weight

The inhibitory effect of *E*. *adenophorum* oil extracts on wet and dry mycelial weights of *P*. *myriotylum* were determined according to the method described by Dikbas et al. [23]. The oil extracts were prepared using DMSO (0.5% v/v) as the initial solvent carrier followed by dilution with potato dextrose broth (PDB) to obtain test concentrations of 40, 60, 80, 100 and 120 μg/ml. Next, a 40 ml liquid medium containing different concentrations of *E*. *adenophorum* oil extracts was placed in each Erlenmeyer flask. Four 4 mm mycelial disks, cut from the periphery of the 2-day-old culture, were added to each flask. The control treatments (without oil extracts) were inoculated using the same procedure. The flasks were then incubated at 28 ± 1°C for 7 days in an incubator shaker. The fungal mycelia were harvested by filtration (separating them from liquid culture) and then washed three times with distilled water. The wet weight of mycelia was determined. The mycelia were then dried at 80°C for 6 hours, and the dry weight of mycelia was determined. Each treatment consisted of three replicates.

### Antifungal effect of *E*. *adenophorum* oil extracts on inoculated ginger

Selected, healthy fresh ginger rhizomes were collected from Shandong Province, China. They were washed in running water, dipped in 70% ethanol for 5 min, and then washed 4 times with sterile water. The surface-sterilized ginger rhizomes were cut into thin slices (2–3 mm). Three concentrations of *E*. *adenophorum* oil extracts (100, 160, 200 μg/ml) were prepared with 0.5% (v/v) DMSO and 0.01% Tween-80. Each ginger slice was dipped separately in the oil extracts for 5 seconds and placed into a sterile Petri dish (9 cm in diameter), then inoculated with *P*. *myriotylum* by placing a 4 mm disc of mycelial material cut from the periphery of 2-day-old culture. All treatments consisted of three replicates with two ginger slices per replicate, and the entire experiment was repeated twice. The controls were prepared in similar manner using a mixture of 0.5% (v/v) DMSO and 0.01% Tween-80 instead of *E*. *adenophorum* oil extracts. All the Petri dishes were incubated at 28 ± 1°C for 4 days and the percentage of infected ginger was observed and recorded.

### Light microscope examination of the effect of *E*. *adenophorum* oil extracts on morphology of *P*. *myriotylum*

In order to investigate the impact of *E*. *adenophorum* oil extracts on the hyphal morphology of *P*. *myriotylum*, a small amount of hyphae was taken from the edges of a colony grown on PDA treated with 80 μg/ml *E*. *adenophorum* oil extracts after ten days of incubation at 28 ± 1°C. The samples were then dipped in sterile water on a glass slide, covered with a glass cover slip, and observed under the microscope (Olympus BX63) at 400x magnification to determine any structural modifications. A control group, cultured without *E*. *adenophorum* oil extracts, was processed using the same procedures. Photographs were taken with computer-attached cellSens^™^ technology (Olympus Corporation, Japan).

### Transmission electron microscopy (TEM) observations

Ten-day-old fungal materials of *P*. *myriotylum* in PDB were amended with 0, 40, 60 and 80 μg/ml *E*. *adenophorum* oil extracts were used for the TEM observation to study the mode of action of oil extracts [24]. The mycelium pellets were treated with 2.5% glutaraldehyde at 4°C, followed by rinsing with 0.1 M phosphate buffer (pH 7.2) and fixed with 1% w/v osmium tetraoxide solution. The fixed samples were rinsed with the same buffer three times. Afterwards, the samples were dehydrated using a series of ethanol solutions in the order of concentration 30, 50, 70, 80, 90, 95 and 100%. After dehydrating and embedding in Spurr’s resin, thin sections were cut and double-stained with uranyl acetate and lead citrate. The grids were examined with a New Bio-TEM H-7500 transmission electron microscope (Hitachi Company, Japan).

## Data analysis

The results were statistically analyzed using SPSS 16.0. All data were reported as mean ± standard error. The data were also analyzed using Duncan’s multiple comparison tests at a significance level of 0.05, following a one-way ANOVA comparison test.

## Results

### Chemical composition of *E*. *adenophorum* oil extracts

GC/MS analysis resulted in the identification of twelve compounds representing 99.15% of the total oil composition (Table 1). The main components were oxygenated sesquiterpenes, such as 10Hβ-9-oxo-agerophorone (37.03%), 10Hα-9-oxo-agerophorone (37.73%) and 9-oxo-10, 11-dehydro-agerophorone (23.41%). Other components such as phytol (0.09%), 1-heptatriacotanol (0.01%) and geranyl linalool (0.45%) were present in smaller amounts.

**Table 1:** Chemical composition of the oil extracts isolated from *Eupatorium adenophorum*.

Number	Retention time (min)	Compound	Identified molecular	% Composition
1	13.93	(-)-Spathulenol	C_15_H_24_O	0.15
2	14.74	1-Heptatriacotanol	C_37_H_76_O	0.01
3	14.82	β-Eudesmol	C_15_H_26_O	0.18
4	15.22	Isoaromadendrene epoxide	C_15_H_24_O	0.03
5	16.52	10Hβ-9-Oxo-agerophorone	C_15_H_22_O_2_	37.03
6	16.75	10Hα-9-Oxo-agerophorone	C_15_H_22_O_2_	37.73
7	17.29	9-Oxo-10,11-dehydro-agerophorone	C_15_H_20_O_2_	23.41
8	17.66	Dihydroxanthin	C_17_H_24_O_5_	0.02
9	19.41	Phytol	C_20_H_40_O	0.09
10	19.69	Dehydroisophytol	C_20_H_38_O	0.01
11	21.11	Geranyl linalool	C_20_H_34_O	0.45
12	21.36	Aromadendrene oxide-(1)	C_15_H_24_O	0.04
			Total	99.15

### Antifungal activity assay

**Fig 1:**
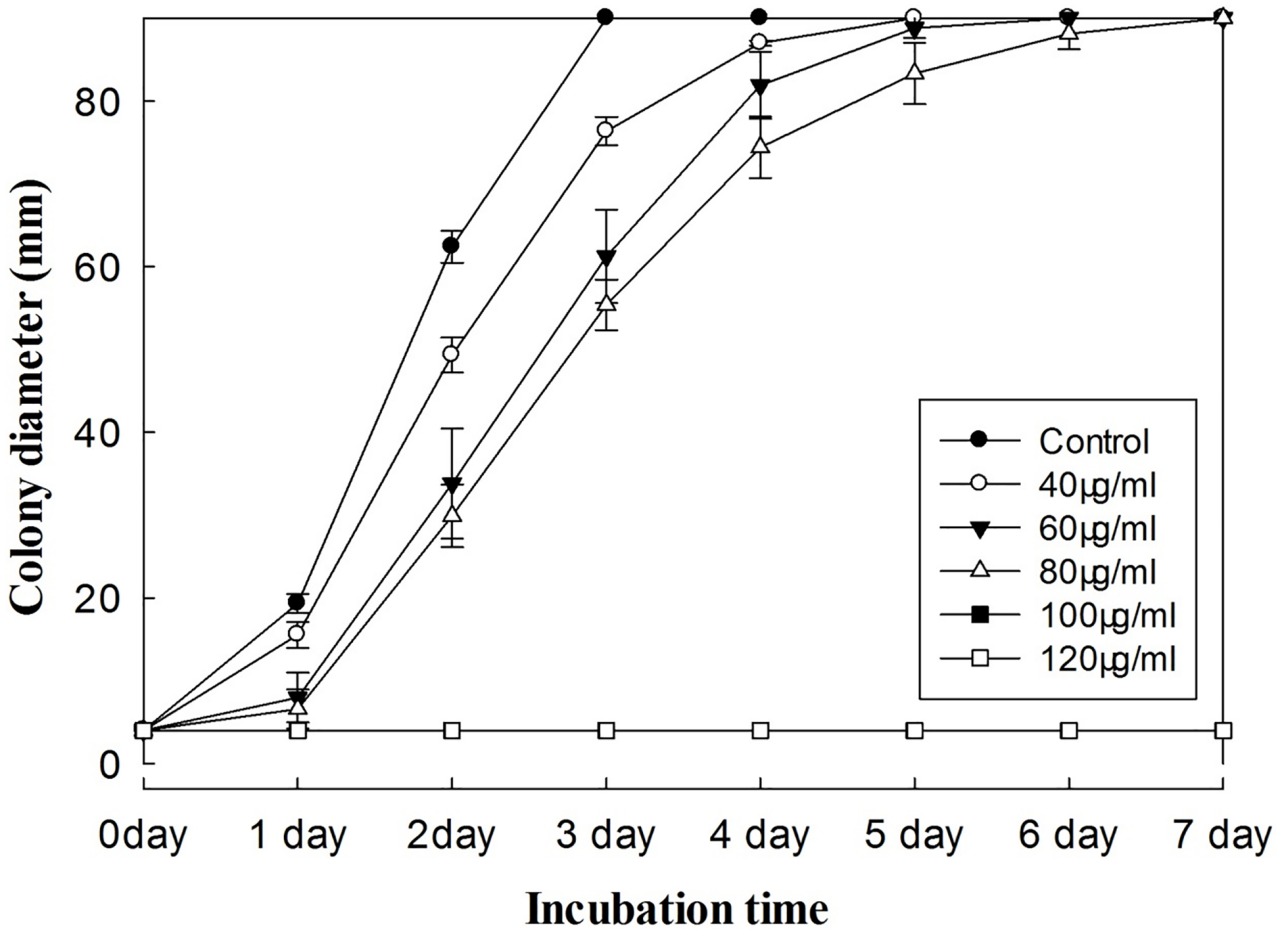
Time-response relationship of the inhibitory effects of increasing concentrations of *E*. *adenophorum* oil extracts on the colony growth of *P*. *myriotylum* on PDA. The response at 100 μg/ml is the same as at 120 μg/ml and is therefore not visible in the Figure. Plates were incubated at a temperature of 28 ± 1°C for 7 days. Values are means (n = 3) ± standard error.

**Fig 2:**
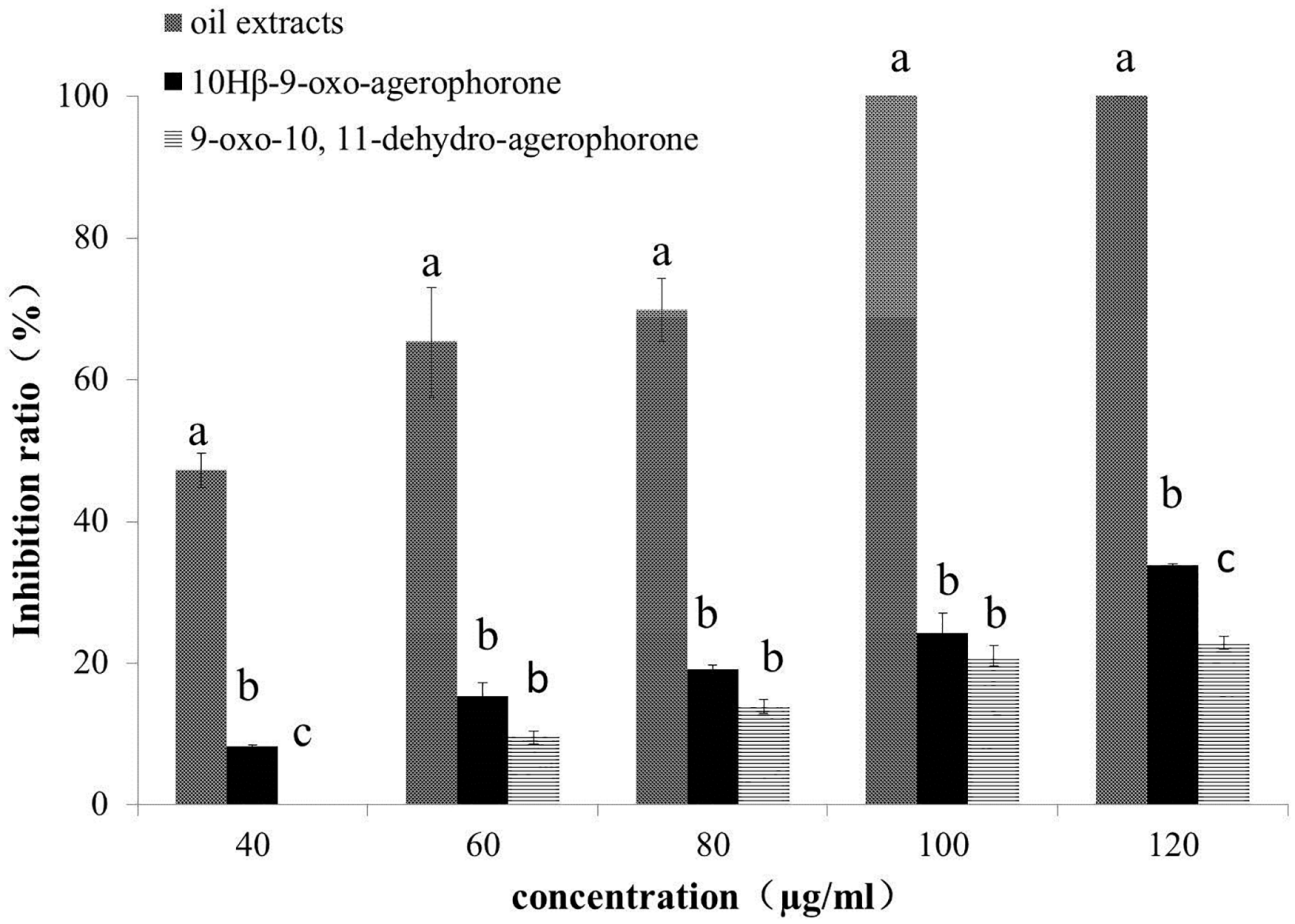
Percentage inhibition ratio of mycelial growth of *P*. *myriotylum* by *E*. *adenophorum* oil extracts and sesquiterpenes at various concentrations (40–120μg/ml). Plates were incubated at 28 ± 1°C for 2 days. Values are means (n = 3) ± standard error. Vertical bars within the same concentration with different letters are significantly different according to ANOVA test (p< 0.05).

The growth of *P*. *myriotylum* treated with *E*. *adenophorum* oil extracts was observed over seven days. The results showed that mycelia growth was reduced proportionately with increasing oil extract concentration (Fig 1). The mycelial growth was complete inhibited at 100 and 120 μg/ml concentrations after seven days of incubation. A concentration of 100 μg/ml of oil extracts was therefore regarded as the minimum inhibitory concentration for *P*. *myriotylum*. The response at 100 μg/ml is the same as at 120 μg/ml and is therefore not visible in Fig 1. The inhibition ratio of oil extracts against *P*. *myriotylum* was determined on the second day of incubation. At that time, mycelial growth was significantly inhibited at concentrations of 40, 60 and 80 μg/ml oil extracts, with reduction percentages of 47.27%, 65.31% and 69.86%, respectively (Fig 2). At the same concentration of oil extracts, 9-oxo-agerophorone and 9-oxo-10, 11-dehydro- agerophorone exhibited relatively poor control efficacy with inhibition of 0% - 33.82%.

### Combined action of *E*. *adenophorum* oil extracts and commercially available fungicides

Table 2 shows the relative growth inhibition of *P*. *myriotylum* to mixtures of *E*. *adenophorum* oil extracts and the synthetic fungicides: azoxystrobin, iprodione, mancozeb and mefenoxam. Importantly, all combinations of the oil extracts and these synthetic fungicides showed substantial synergistic effects on the mycelial growth of *P*. *myriotylum*. The synergism ratios (SR) ranged from 2.07 to 33.29 for the tested combinations of oil extracts and fungicides. Mancozeb alone (20 μg/ml) and a very low concentration of oil extracts alone (5 μg/ml) inhibited growth by 80.47% and 6.95%, respectively. However, the same concentrations of fungicide and oil extracts mixed together completely inhibited mycelial growth. Complete growth inhibition of *P*. *myriotylum* was also observed using a mixture of 100 μg/ml iprodione + 50 μg/ml oil extracts, compared with only 22.52% reduction in mycelial growth when 100 μg/ml iprodione was used alone. The oil extracts appear to synergise the effects of relatively low concentrations of mancozeb and iprodione fungicides against *P*. *myriotylum*.

**Table 2:** Inhibitory effect of various concentrations of oil extracts from the leaves of *Eupatorium adenophorum* and commercial fungicides (two-way mixtures) on radial growth of *Phythium myriotylum* on PDA medium.

Treatment (μg/ml)	Growth inhibition (%)	SR	Treatment (μg/ml)	Growth inhibition (%)	SR
Oil extracts 5	6.95 ± 2.06	-	Oil extracts 40	47.27 ± 2.43	-
Oil extracts 10	9.97 ± 1.86	-	Oil extracts 50	51.38 ± 1.49	-
Mefenoxam 0.625	89.94 ± 2.23	-	Iprodione 100	22.52 ± 4.57	-
Mefenoxam 1.25	94.28 ± 0.24	-	Iprodione 200	37.28 ± 6.17	-
Oil extracts 5 + Mefenoxam 0.625	95.55 ± 1.66	15.29	Oil extracts 40 + Iprodione 100	50.55 ± 2.05	4.75
Oil extracts 5 + Mefenoxam 1.25	95.92 ± 1.85	14.62	Oil extracts 40 + Iprodione 200	81.95 ± 12.25	4.65
Oil extracts 10 + Mefenoxam 0.625	97.37 ± 0.23	10.86	Oil extracts 50 + Iprodione 100	100.00 ± 0.00	8.64
Oil extracts 10 + Mefenoxam 1.25	96.35 ± 0.84	10.25	Oil extracts 50 + Iprodione 200	86.68 ± 6.67	4.52
Mancozeb 10	24.90 ± 3.45	-	Azoxystrobin 50	82.35 ± 2.53	-
Mancozeb 20	80.47 ± 19.53	-	Azoxystrobin 100	84.84 ± 2.83	-
Oil extracts 5 + Mancozeb 10	57.59 ± 7.29	33.29	Oil extracts 40 + Azoxystrobin 50	95.00 ± 1.76	2.44
Oil extracts 5 + Mancozeb 20	100.00 ± 0.00	17.86	Oil extracts 40 + Azoxystrobin 100	92.69 ± 1.33	2.31
Oil extracts 10 + Mancozeb 10	53.91 ± 9.34	21.74	Oil extracts 50 + Azoxystrobin 50	91.23 ± 2.87	2.16
Oil extracts 10 + Mancozeb 20	84.87 ± 15.12	10.57	Oil extracts 50 + Azoxystrobin 100	90.14 ± 1.41	2.07

All data are the means of three experiments ± standard deviation. SR = O_bs_ / E_xp._

### Antifungal effect of *E*. *adenophorum* oil extracts on wet and dry mycelial weight

The inhibitory effects of *E*. *adenophorum* oil extracts on wet and dry weights of mycelia were tested in PDB medium (Table 3). Various concentrations of the oil extracts were found to be effective in inhibiting *P*. *myriotylum* to form or increase biomass. The biomass of *P*. *myriotylum* was reduced to about half of that of the control at concentrations of 60 μg/ml oil extracts. The oil extracts completely inhibited mycelial growth and biomass formation of *P*. *myriotylum* at 120 μg/ml.

**Table 3:** Wet and dry mycelium weight of *P*. *myriotylum* in liquid medium following the addition of various concentrations of *E*. *adenophorum* oil extracts.

Treatments (μg/ml)	Wet mycelium weight (mg)*	Dry mycelium weight (mg)*
0	1585.07 ± 62.85 a	95.85 ± 0.59a
40	1446.30 ± 285.98 a	80.35 ± 22.88ab
60	704.53 ± 186.21b	59.05 ± 13.07bc
80	294.83 ± 61.78bc	25.68 ± 5.51cd
100	112.47 ± 85.59c	6.21 ± 6.98d
120	0 ± 0c	0 ± 0d

*Values are means ± standard error; data in columns with different letters are statistically different according to Duncan’s multiple comparisons tests at *p* < 0.05; each treatment consisted of three replicates.

### Antifungal effect of *E*. *adenophorum* oil extracts on ginger inoculated with *P*. *myriotylum*

**Fig 3:**
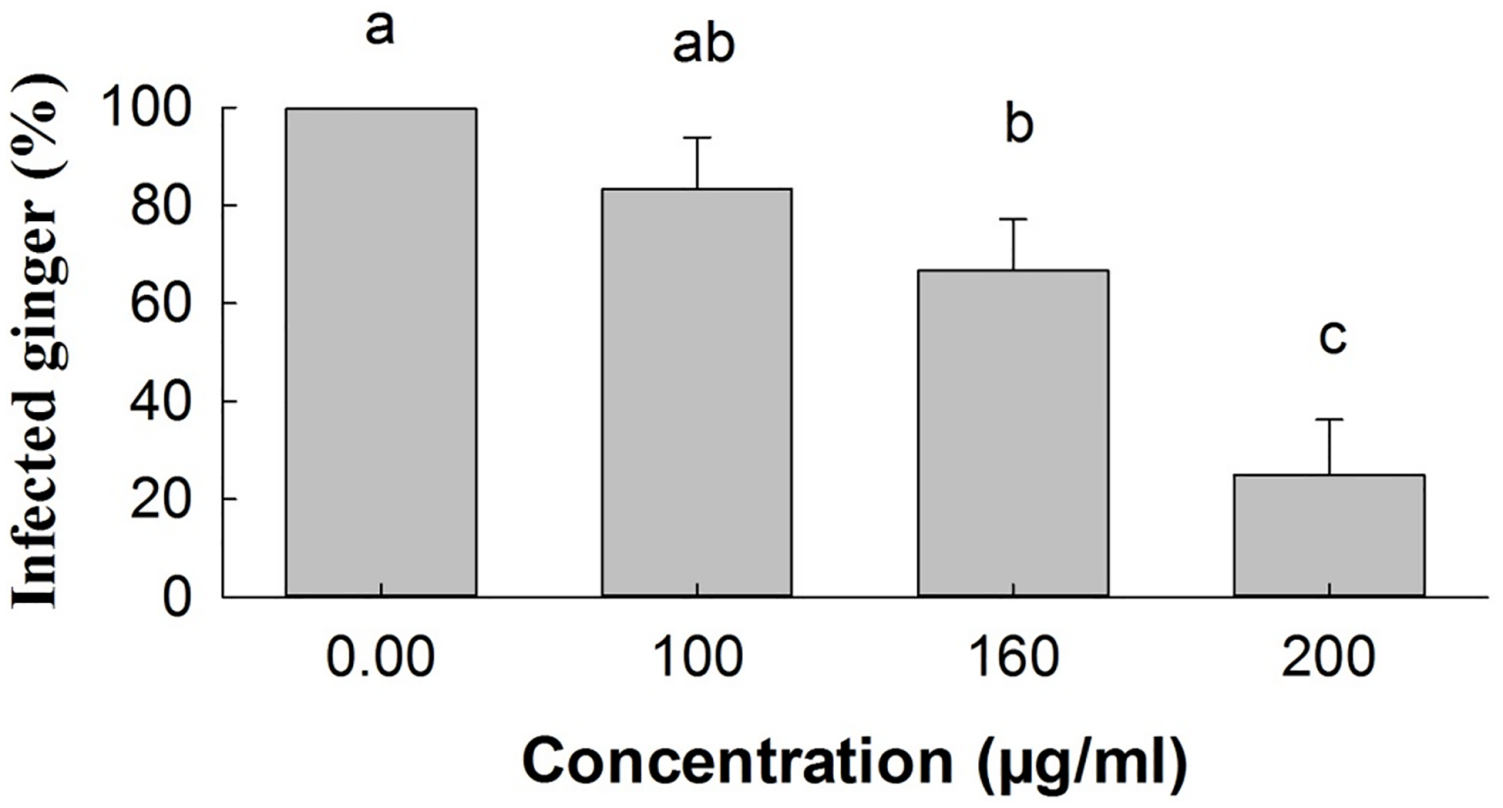
Percentage of detached ginger rhizomes showing disease symptoms as a result of infection with *P*. *myriotylum* after first being treated with oil extracts from *E*. *adenophorum* at various concentrations. The observations were made 4 days after inoculation. Values are means (n = 3) ± standard error. Vertical bars with different letters are significantly different according to ANOVA test (p< 0.05).

**Fig 4:**
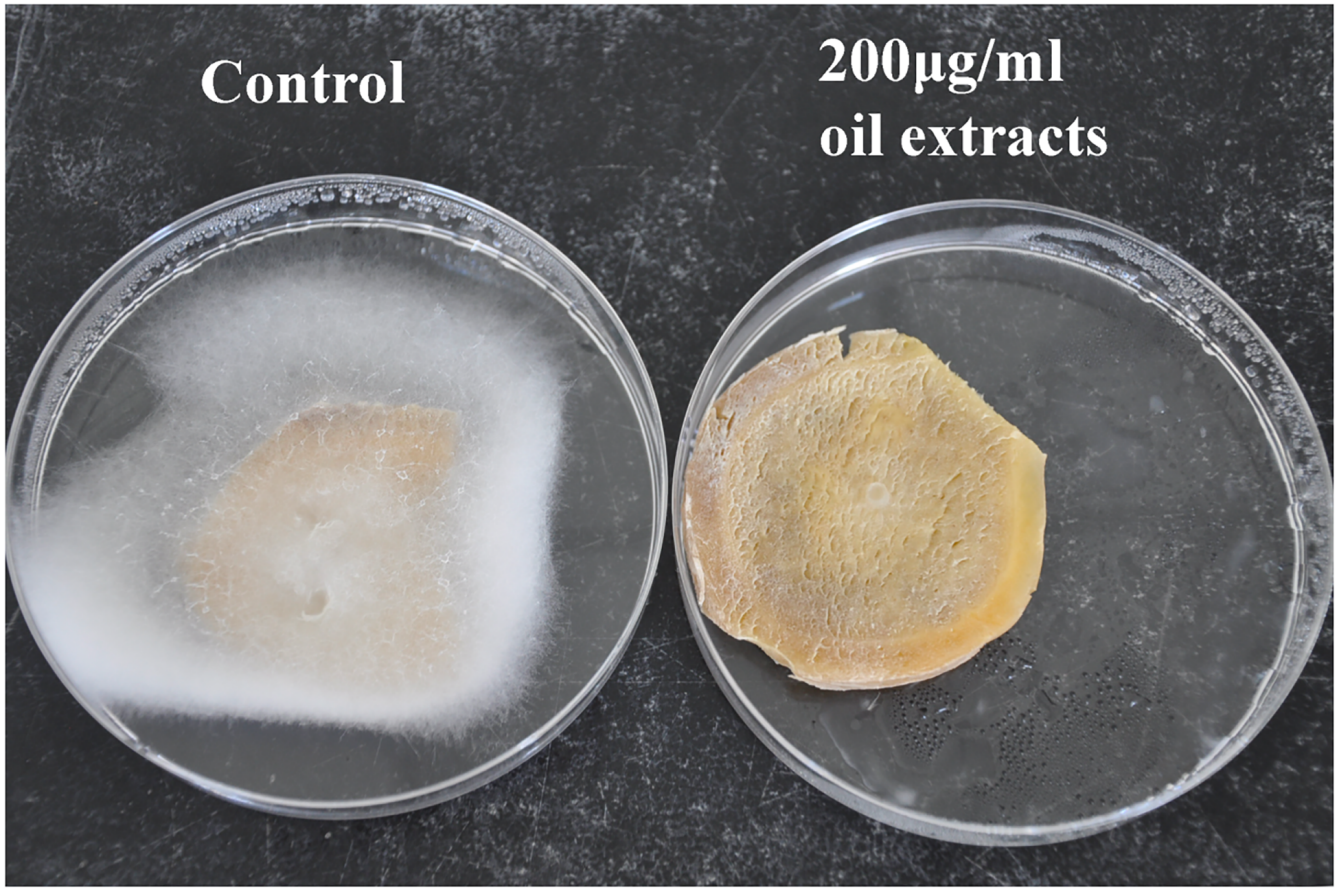
Decayed ginger (left) that had been inoculated with the pathogen *P*. *myriotylum* (control), and healthy ginger (right) as a result of the treatment by oil extracts (200 μg/ml concentration) applied before pathogen inoculation, and kept at 28 ± 1°C for 4 days.

Fig 3 shows the results of tests on the antifungal activity of *E*. *adenophorum* oil extracts on ginger rhizome samples inoculated with *P*. *myriotylum* and incubated at 28 ± 1°C. The percentage of detached ginger rhizomes showing disease symptoms as a result of infection with *P*. *myriotylum* was reduced after first being treated with oil extracts from *E*. *adenophorum* at various concentrations The percentage of infected ginger was reduced significantly (P<0.05) compared with the control groups in samples that were treated with 160 and 200 μg/ml of oil extracts before being exposed to the pathogen. Ginger samples that were not treated with *E*. *adenophorum* oil extracts became completely infected with *P*. *myriotylum*. Fig 4 shows the results 4 days after inoculation with *P*. *myriotylum*. Decayed ginger (left) that was inoculated with the pathogen *P*. *myriotylum* (control), and healthy ginger (right) that had oil extracts (200 μg/ml concentration) applied before the pathogen was inoculated.

### Light microscope examination of the effect of oil extracts on morphology of *P*. *myriotylum*

**Fig 5:**
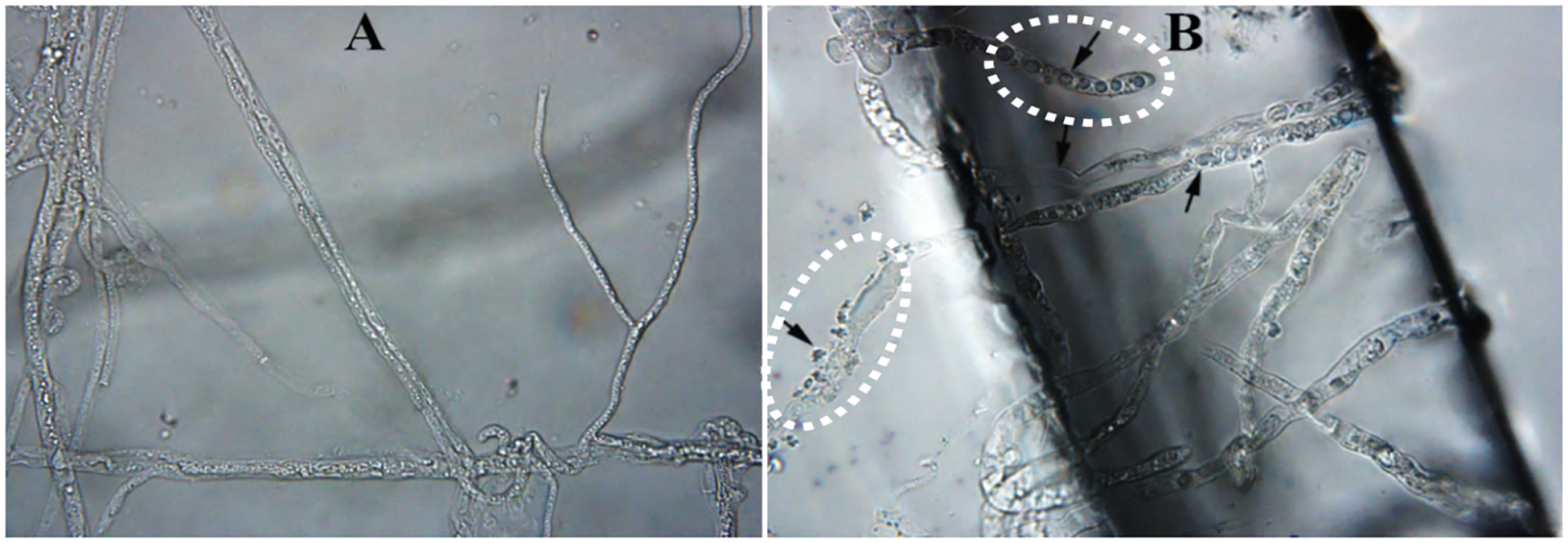
Light microscope observations of *P*. *myriotylum* mycelia. A. Untreated (400x); B. Treated with 80 μg/ml of oil extracts from *E*. *adenophorum* (400x).

The response of *P*. *myriotylum* to *E*. *adenophorum* oil extracts was observed using a light microscope at 400x magnification. *P*. *myriotylum* mycelia treated with 80 μg/ml oil extracts were compared with an untreated control. Compared with the control, hyphae exposed to the oil extracts were substantially modified (Fig 5). Untreated mycelia developed regular and homogeneous hyphae (Fig 5A). Compared with the control, mycelia treated with oil extracts produced larger diameter hyphae; a more heterogeneous distribution of the cytoplasmic matrix; more cytoplasmic granulation; less cytoplasmic matrix; and more decay of the cell wall (Fig 5B).

### Transmission electron microscopy (TEM) observations

**Fig 6:**
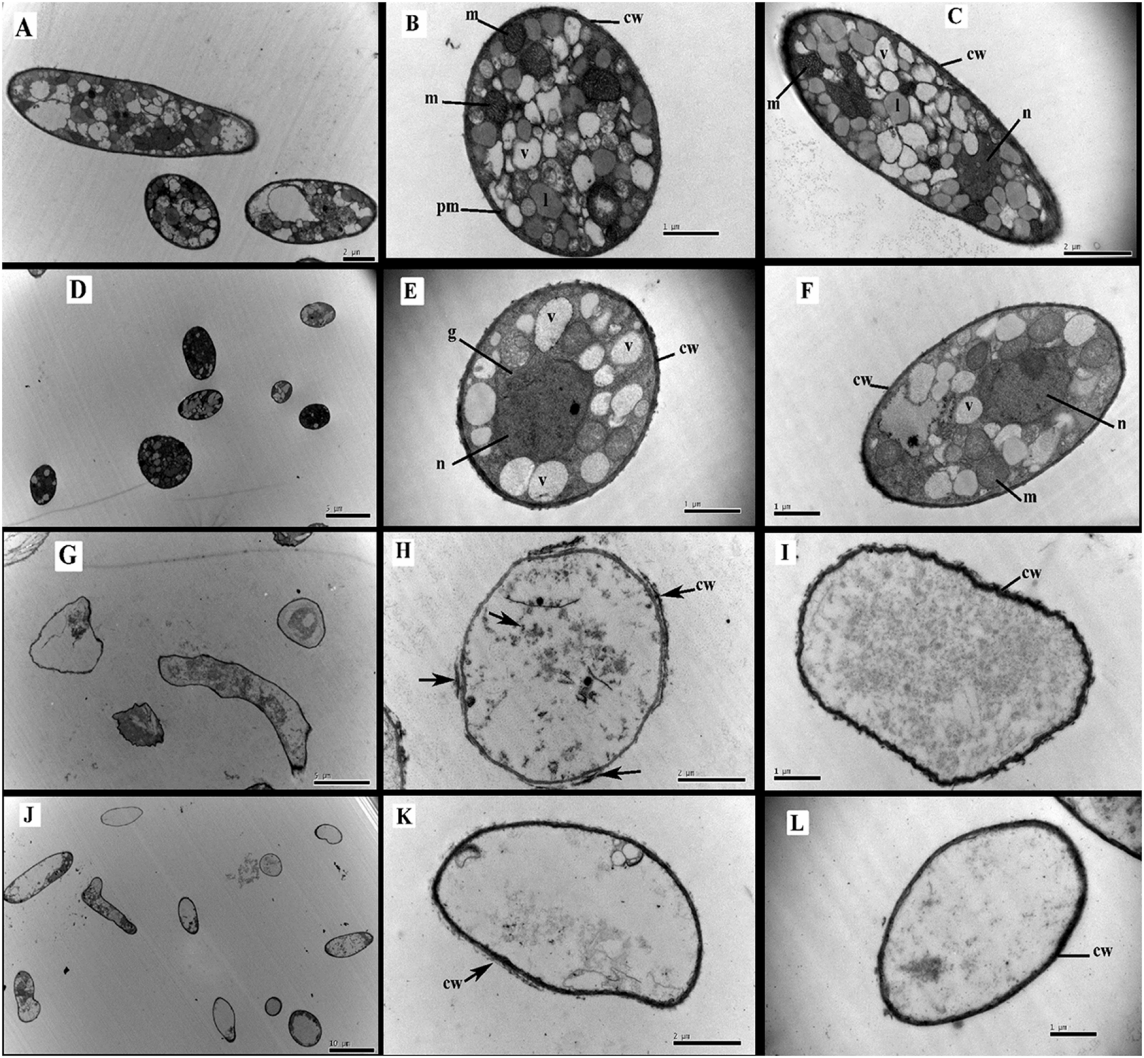
Transmission electron microscopy illustrating the effect of oil extracts from *E*.*adenophorum* on the ultrastructural aspects of the pathogen *P*. *myriotylum*. A-C: Control; D-F: treated with 40 μg/ml oil extracts; G-I: treated with 60 μg/ml oil extracts; J-L: treated with 80 μg/ml oil extracts. cw = cell wall; g = golgi apparatus; pm = plasma membrane; l = liposome; m = mitochondria; n = nucleus; v = vacuole.

Ultrastructural changes to *P*. *myriotylum* as a result of exposure to *E*. *adenophorum* oil extracts were observed using TEM. The control mycelia of *P*. *myriotylum* grown in the absence of oil extracts showed the cell wall to be uniform; the cytoplasmic matrix was abundant; and organelle-rich cytoplasm was present that included mitochondria (m), golgi apparatus (g), vacuole (v), liposome (l) and nucleus (n) (Fig 6). All of those organelles have normal and uniform structures (Fig 6A–6C). In the presence of oil extracts at 40 μg/ml, the most conspicuous ultrastructural change observed was the lack of liposomes in the cytoplasmic matrix. The vacuoles, nucleus and mitochondria had the same appearance as the control hyphae, except there were fewer of them. At a higher concentration of 60 μg/ml, the oil extracts caused more obvious ultrastructural alterations: The cells were abnormally shaped, the cytoplasmic organelles were no longer present, cytoplasmic matrixes were absent, the cell wall was thinner and cell wall debris was observed (Fig 6G–6I). At a concentration of 80 μg/ml oil extracts, the cell ultrastructure damage was even more apparent as organelles and most cytoplasmic inclusions were completely absent, leaving only mostly empty cavity cells (Fig 6J–6L).

## Discussion

Improved living standards have resulted in the public demanding greater food safety. In the process of searching for plant protection agents with reduced risks to food safety, there has been an increasing attention on secondary metabolites of plants. *E*. *adenophorum* has been used by the Tamang tribes as an herbal medicine for treating fever and insomnia [25]. Previous studies showed that *E*. *adenophorum* essential oil is rich in terpenes [14]. This class of compounds defends many species of plants, animals and microorganisms against predators, pathogens and competitors [26]. Terpenes in *E*. *adenophorum* therefore show great promise as plant protection agents.

This study used methanol and ethyl acetate to extract sesquiterpenes from *E*. *adenophorum*. Sesquiterpenes accounted for 98.57% of the oil extracts isolated using this method. A previous study that used a hydrodistillation method to extract sesquiterpenes in inflorescence oil and root oil of *E*. *adenophorum* reported a different composition of inflorescence and root oils [14]. The use of different extraction methods might account for differences in the composition of the oils in the sesquiterpenes.

Previous studies by Kundu et al. [13] and Ouyang et al. [27] detected 9-oxo-agerophorone (10Hα and 10Hβ) and 9-oxo-10, 11-dehydro-agerophorone in ethyl acetate extract of *E*. *adenophorum* leaves, but this two compounds were given different names (cadinan-3-ene-2,7-dione and cadinan-3,6-diene-2,7-dione) by Kundu et al. [13]. The oral toxicity of 9-oxo-agerophorone (10Hα and 10Hβ) and 9-oxo-10, 11-dehydro-agerophorone on mice was reported to be low [27]. However, these compounds were effective against *Rhizoctonia solani*, *Sclerotium rolfsii*, *Fusarium oxysporum* and *Macrophomina phaseolina*, with EC_50_ values ranging from 89.74 μg/ml to 320 μg/ml [13]. Moreover, the antifungal activity of sesquiterpenes on *P*. *myriotylum* had not been reported.

Our study indicated that *E*. *adenophorum* oil extracts have a marked inhibitory effect on *P*. *myriotylum*. The mycelial growth was completely inhibited when exposed to oil extracts of 100μg/ml. The antifungal activity of *E*. *adenophorum* oil extracts might be attributed to the presence of sesquiterpenes, because 9-oxo-agerophorone and 9-oxo-10, 11-dehydro-agerophorone are known to be strong antifungal compounds [13, 19, 28]. But the oil extracts have greater antifungal activity, possibly due to the effect of sesquiterpenes being synergised in the presence of other compounds.

Our laboratory bioassays demonstrated a strongly synergistic effect on *P*. *myriotylum* growth when *E*. *adenophorum* oil extracts were mixed with commonly-used synthetic fungicides (mefenoxam, mancozeb, iprodione or azoxystrobin). Moreover, the antifungal activity of oil extracts was higher than that of iprodione. The precise mechanism of this synergistic inhibition of *P*. *myriotylum* growth is not known. It is likely, however, that the synergistic effect is due to the components of the mixture targeting different sites in *P*. *myriotylum* mycelial cells [29]. Mefenoxam inhibits ribosomal RNA (rRNA) biosynthesis [30], while mancozeb inhibits pyruvic acid oxidation. The antifungal activity of iprodione is related to its intervention effects on protein kinase in the signal transduction pathway. The mechanism of action of *E*. *adenophorum* oil extracts is not clear, but the mode of action of its major component—terpene—is speculated to involve membrane disruption by lipophilic compounds [31]. The low-molecular weight and highly lipophilic compounds can easily diffuse across cell membranes to induce biological reactions [32]. The synergy could conceivably be the result of a general increase in stress when different cellular processes are attacked simultaneously [29]. Or, to be more precise, membrane disruption may allow a greater quantity of synthetic fungicides to reach the target sites successfully.

This study also showed that the *E*. *adenophorum* oil extracts reduced the biomass of the mycelium of *P*. *myriotylum* in a dosage-response manner. *P*. *myriotylum* was completely inhibited at 120 μg/ml in a liquid medium and by 100 μg/ml in a solid medium. This indicated that the oil extracts may be more effective in a solid rather than in a liquid medium.

*In vitro* studies on *E*. *adenophorum* oil extracts indicate their potential as a suitable antifungal agent against *P*. *myriotylum*. *In vivo* studies will be necessary to determine its efficacy as a botanical pesticide for the control of root rot in commercially-produced vegetables. The present results clearly demonstrate that *E*. *adenophorum* oil extracts significantly reduce decay in artificially inoculated ginger. However, higher concentrations of the oil extracts may be required in the field where pathogen growth may be favored by better nutritional and moisture conditions than those in the laboratory [33].

Although previous studies have highlighted the antifungal activity of plant extracts or essential oils from *E*. *adenophorum*, few have demonstrated morphological and ultrastructural changes to fungi. Light microscope observations of the microstructure of *P*. *myriotylum* revealed several mechanisms by which *E*. *adenophorum* oil extracts effect this fungal pathogen. Degenerative changes in cell inclusion and hyphal morphology were commonly observed, including cytoplasm granulation, lack of cytoplasm, increased hyphal diameter and cell wall decay (Fig 5). The changes indicated that the cell wall, cell membrane and cytoplasm may be the targets for *E*. *adenophorum* oil extract components. However, not all of these cellular components are separate targets. Some targets may be affected as a consequence of another mechanism being activated by the oil extract components [34]. It is likely that attacks at multiple cellular targets will eventually lead to cell death.

*E*. *adenophorum* oil extracts had caused morphological and ultrastructural changes within *P*. *myriotylum* in a concentration-dependent manner. Results from the TEM are in agreement with light microscope observations. Oil extracts eliminated almost entirely cytoplasm and cellular organelles from hyphae. This suggested that the mode of antifungal activity of oil extracts of *E*. *adenophorum* is a result of the oil extracts targeting the fungal plasma membrane and endomembrane systems. Somewhat similar phenomena were observed in TEM studies by Tian et al. using *Cinnamomun jensenianum* essential oil on *Aspergillus flavus*, which is an oil abundant in terpenes [35]. However, it was not clear whether *E*. *adenophorum* oil extracts destroy the membrane system directly by preventing membrane formation or indirectly by inducing new substance which damaged membrane integrity.

This work has shown that oil extracts from *E*. *adenophorum* possess fungitoxic activities that inhibit the growth of *P*. *myriotylum*, thereby increasing the possibility for using these oil extracts to control *P*. *myriotylum* in commercial crops in the future. The use of these oil extracts will also turn *E*. *adenophorum* from a weed to a valuable resource. Further studies are required to determine the stability of these oil extracts in the field and their phytotoxicity to a range of commercial crops.
